# Piloting a cancer awareness app across six European countries: a pre-post study

**DOI:** 10.3389/fpubh.2025.1648212

**Published:** 2025-09-29

**Authors:** Furqan Ahmed, Silvana Melissa Romero Saletti, Erica D’Souza, Carolina Espina, David Ritchie, Ana Molina Barceló, Marina Pinto Carbó, Paula Romeo Cervera, Teresa Seum, Hermann Brenner, Stephan Van den Broucke, Maria Krini, Cristiana Fonseca, Patricia Pinto, Diana Krivic, Helena Ros Comesana, Wendy Yared, Rebekka Wiersing, Hajo Zeeb, Tilman Brand

**Affiliations:** ^1^Department of Prevention and Evaluation, Leibniz Institute for Prevention Research and Epidemiology – BIPS, Bremen, Germany; ^2^Psychological Sciences Research Institute (IPSY), Université Catholique de Louvain, Louvain-la-Neuve, Belgium; ^3^Environment and Lifestyle Epidemiology Branch, International Agency for Research on Cancer (IARC-WHO), Lyon, France; ^4^Cancer and Public Health Research Unit, The Foundation for the Promotion of Health and Biomedical Research of Valencia Region (FISABIO), Valencia, Spain; ^5^Division of Clinical Epidemiology and Aging Research, German Cancer Research Center (DKFZ), Heidelberg, Germany; ^6^Pagkyprios Syndesmos Karkinopathon kai Filon 1986 (PASYKAF), Nicosia, Cyprus; ^7^Liga Portuguesa Contra o Cancro (Portuguese League Against Cancer, LPCC), Lisboa, Portugal; ^8^Association of Slovenian Cancer Societies (ASCS), Ljubljana, Slovenia; ^9^Association of European Cancer Leagues (ECL), Brussels, Belgium; ^10^Health Sciences Bremen, University of Bremen, Bremen, Germany

**Keywords:** cancer prevention, mobile health, digital health literacy, usability, European Union, pre-post study

## Abstract

**Background:**

Cancer remains a significant public health challenge in Europe, accounting for over 22% of global cancer cases. Mobile health applications may help to increase the awareness of cancer risks and preventive behaviors. However, usability barriers and disparities in digital health literacy (DHL) may limit their impact.

**Objective:**

This study evaluated the usability of the EU Mobile App for Cancer Prevention and changes in cancer awareness associated with use across six European countries (Cyprus, Germany, Hungary, Portugal, Slovenia, Spain), focusing on variations by DHL and sociodemographic factors.

**Methods:**

A pre-post design was employed, combining pre- and post-usage surveys (N = 328 pre; n = 77 post). Participants interacted with the app for 7 days. Usability was assessed via the System Usability Scale (SUS), cancer awareness via an 18-item quiz, and DHL using the eHEALS tool. Descriptive statistics summarized key variables, while t-tests and ANOVAs assessed group differences in usability and cancer awareness outcomes.

**Results:**

A total of 328 participants completed the pre-usage survey, with 77 proceeding to the post-usage phase; the primary reason for dropout was technical difficulties. The app’s overall usability was rated as moderate (Mean = 62.56 on the SUS) and did not differ significantly across demographic or usage subgroups. Cancer awareness scores improved from 50.45 to 53.31 (*p* < 0.001) showing significant moderate improvement, particularly among those with lower DHL and higher education. We observed no dose response relationship between self-reported app usage (frequency or session duration) and changes in cancer awareness.

**Conclusion:**

This pilot study demonstrates that the EU Mobile App for Cancer Prevention can enhance cancer awareness, while currently the usability was judged to be moderate. Targeted refinements in navigation, setup procedures, and content tailoring for low-DHL users are essential to improve engagement and ensure equitable reach.

## Background

Cancer is a major global health issue, with nearly 20 million new cases and 9.7 million deaths reported in 2022 alone (1). Europe comprises 22% of global cancer incidence and 20% of cancer-related mortality. This impact is attributed to an aging population and lifestyle choices. Projections indicate that by 2050, cancer incidence in Europe could rise by 25%, underscoring the urgent need for effective prevention strategies (2–4).

Approximately 40% of cancers are preventable through lifestyle modifications such as quitting smoking, improving dietary habits, reducing alcohol consumption, and modifiable environmental exposures such as reducing air pollution and prevention cancer-causing infections (5). The European Code Against Cancer (ECAC)—an initiative of the European Union, developed in 1987 and fourth edition updated in 2014—provides 12 evidence-based recommendations for cancer risk reduction (6). These guidelines are disseminated through partnerships with health organizations and cancer leagues, supporting regional prevention efforts. Europe’s Beating Cancer Plan (EBCP), launched in 2021, focuses on leveraging digital innovation to enhance cancer prevention, particularly in underserved populations (7). A pivotal component of this plan is the EU Mobile App for Cancer Prevention, designed to improve access to ECAC recommendations via mobile technology (8).

While digital health tools offer significant potential, they also present challenges, particularly regarding digital health literacy (DHL)— the ability to seek, find, understand, and appraise health information from electronic or digital sources and to apply the knowledge gained to addressing or solving a health problem (9–11). Approximately 10% of Europeans lack basic health literacy, with the most affected groups being older adults, rural residents, and marginalized communities (12). Poor DHL may be associated with delayed or non-participation in screening programmes, non-compliance with preventive guidelines, and mistrust of digital resources, which exacerbates health disparities (13). If digital health technologies are not intentionally designed with accessibility and usability in mind, they risk excluding populations who are often most affected by health inequities—specifically those bearing the highest burden of disease and social risk factors.

Mobile health (mHealth) apps can address these gaps by providing personalized education, behavior change support, and evidence-based preventive information. Evidence indicates mHealth interventions can improve outcomes in several domains (e.g., smoking cessation; management of noncommunicable diseases), though effects vary by dose, duration, and engagement (14–16). The effectiveness of digital health interventions relies on their usability. Nevertheless, many applications do not achieve the required system usability standards, which hinders equitable access and use, especially considering the diverse levels of DHL among populations (17).

With global cancer incidence projected to reach 35 million new cases annually by 2050, innovations such as the EU Mobile App for Cancer Prevention are increasingly critical (1). Launched in 2022, the Boosting the Usability of the EU Mobile App for Cancer Prevention (BUMPER) project evaluates both the app’s usability and its impact across European countries (8). By examining the interplay between usability and DHL in prevention tools, the project seeks to inform a redesigned EU Mobile App that aligns with the equity goals of the EBCP.

## Aim of the study

This study assessed the EU Mobile App for Cancer Prevention’s usability and impact via a pilot trial in six European countries. It investigated differences in user experience and perceived usability based on DHL and sociodemographic factors. The main research questions were:

1. What was the perceived usability of the EU Mobile App for Cancer Prevention among users?
2. Does using the app increase users’ awareness of cancer prevention and associated risk factors?
3. How do DHL and sociodemographic factors (age, gender, education) affect users’ perceptions of app usability and awareness outcomes?

## Materials and methods

### Study design and setting

For this pilot trial, a pre-post approach was employed, integrating quantitative surveys (before and after app usage), consistent with prior usability/effectiveness pilots in mHealth, where brief exposure and pre-post assessments are used to gauge feasibility and short-term knowledge change (18, 19). Data was collected online using Limesurvey across the six participating European countries from July to September 2024 (20).

### App description and development

The EU Mobile App for Cancer Prevention features an intuitive, tab-based interface with four sections (Dashboard, Goals, Learning, Profile) for tracking behaviors, setting and monitoring prevention goals, accessing ECAC-aligned educational modules, and personalizing reminders. Content adapts to each user’s demographics and health inputs, with direct links to the “12 ways to reduce your cancer risk.” As part of the BUMPER project, the app was developed in collaboration with the developer through co-design, internal review, testing wireframes, a 7-day pilot test, and partner feedback, resulting in a refined prototype. Table 1 shows the App design.

**Table 1:** Overview of the EU mobile app for cancer prevention design.

Section/Tab	Core elements	Description
Dashboard	Personalized greeting Progress bar Daily log (7-day view)	Greets user by name, shows real-time progress toward goals, and lets users record behaviors (e.g., tobacco use, activity) with a single tap over the past week.
Goals	Suggested / Active / Completed filters Icons for each goal Quantifiable targets (e.g., <10 cigarettes/day, −10.4 kg) Progress visualization	Organizes behavior-change objectives by status; each goal is iconified, has a specific numeric target, and shows progress at a glance.
Learning	“Discover” topic cards Prevention themes (smoking cessation, healthy weight, etc.) Direct links to ECAC’s “12 ways to reduce your cancer risk”	Provides evidence-based educational modules aligned with European guidelines; tapping a topic opens official ECAC recommendations.
Profile	Personal data inputs (age, sex, health info) Settings & preferences	Stores user demographics and self-reported health data to tailor content delivery and reminder schedules for maximum relevance.

### Participant eligibility and recruitment

Participants were eligible if they were adults (≥18 years) residing in one of the six participating countries—Portugal, Spain, Slovenia, Germany, Hungary, and Cyprus. Due to online advertisements and involvement of BUMPER consortium members, a small number or participants from other countries were also included. Participants needed to have access to an internet-enabled smartphone (Android or iOS) and be able to communicate in either English or their local language. Recruitment efforts incorporated a combination of digital outreach strategies, involving collaborations with local health organizations, social media campaigns, partnerships with community cancer leagues, and participant recruitment through platforms like Prolific (21). These diverse methods ensured a broad and varied participant base encompassing different sociodemographic groups. Exclusion criteria included incomplete pre-usage surveys or failure to install the app.

### Data collection procedures

The study employed a two-phase sequential design to evaluate the usability of the mobile application and changes in cancer awareness. Data collection integrated validated instruments and structured surveys across pre-usage, active app engagement, and post-usage stages. Online surveys were developed in Limesurvey to collect data for pre-post usage assessment and to obtain reasons from participants who did not respond to the post-usage survey (21). The survey was administered in English and in local languages (German, Hungarian, Portuguese, Slovenian, Spanish, Greek). Items were translated initially by using DeepL Pro (22) and then reviewed by native speaking investigators for any discrepancies and for clarity. Where validated translations existed (e.g., eHEALS), these were used or adapted with minor changes. Formal psychometric validation across countries was beyond the scope of this pilot.

#### Pre-usage survey

Participants (N = 328) initially completed an online survey capturing demographic, health, and baseline DHL data. Demographic variables included age, gender (male, female, and other), country of residence, and education level. Health-related information encompassed self-reported medical conditions, personal cancer history, and family cancer history. DHL was assessed using the eHealth Literacy Scale (eHEALS), an 8-item instrument scored from 8 to 40, with higher scores indicating greater perceived ability to seek, evaluate, and apply health information from digital sources (23). Upon survey completion, participants received instructions to install the EU Mobile App for Cancer Prevention on their Android or iOS devices. Baseline cancer awareness knowledge was assessed at this stage (see post usage section for details).

#### App usage phase

As this was a pilot usability study and the final version of the app was not yet available on the Apple App Store or Google Play Store, participants were provided a direct download link to a trial version of the app. Android users received an APK file, while iOS participants were sent a link (e.g., via TestFlight) to install the beta version. Clear, step-by-step instructions on how to install the app were drafted and sent via email, and following completion of the pre-survey, participants received three additional invitations/reminders to complete the installation process. Participants who successfully installed the app engaged with its features for at least seven consecutive days.

#### Post-usage survey

After 7 days of app use, participants were sent up to three invitations/reminders to complete a follow-up survey evaluating usability and cancer awareness. A subset of participants (n = 77) completed the survey. The System Usability Scale (SUS)—a 10-item validated tool—was administered to measure perceived usability (24). Responses were recorded on a 5-point Likert scale (1 = Strongly Disagree, 5 = Strongly Agree), with total scores standardized to a 0–100 scale (scores ≥68 denoted above-average usability).

Cancer awareness was assessed using an 18-item quiz adapted from the Cancer Research UK co-developed Cancer Awareness Measure (CAM) (25). This tool evaluated knowledge of risk factors, prevention strategies, and help-seeking behaviors through Likert-scale and categorical questions. Only the questions pertaining to cancer prevention from the CAM were used to evaluate awareness levels. Total scores ranged from 11 to 68, with higher scores reflecting greater awareness (see Supplementary File 1 for detailed scoring and item descriptions). App usage (session frequency and typical session duration) was self-reported in the post-usage questionnaire as telemetry was unavailable in the beta build. Additionally, after the post-usage data collection, a separate questionnaire was sent to those who did not respond to the follow-up survey to understand their reasons for non-participation (Supplementary File 1).

### Data analysis

#### Quantitative analysis

Quantitative analyses were conducted using Python 3.9 with libraries including pandas (v1.3.5) for data preprocessing (such as age calculation and education categorization), SciPy (v1.7.3) and statsmodels (v0.13.2) for hypothesis testing, and matplotlib (v3.5.1) and seaborn (v0.11.2) for visualizations (26). Descriptive statistics (means, standard deviations, medians, frequencies, percentages) summarized demographics, health characteristics, DHL scores, SUS metrics, and app usage patterns. Key variables were categorized as follows:

- Age: Young Adults (18–40 years), Middle-Aged Adults (41–60 years), Older Adults (≥61 years).
- Education: Low (primary/secondary education) vs. High (vocational training, bachelor’s/master’s/doctoral degrees).
- DHL: Median-split into Low (≤29) and High (>29) based on eHEALS scores.

Using the eHEALS tool, DHL was measured as a baseline (pre-usage), then recalculated (not measured) for participants who continued in the post-usage phase. Independent-samples t-tests assessed differences in SUS scores across binary groups (gender, education level, DHL categories), while one-way ANOVAs evaluated differences across multi-group variables (age, country, app usage frequency, and session duration). For cancer awareness scores, paired t-tests compared pre- and post-usage changes, and subgroup differences were analyzed using independent t-tests (gender, DHL) and ANOVAs (27). Country level comparisons of cancer awareness were not undertaken because the post usage sample was small and unevenly distributed across six EU countries; we restricted stratification to individual-level factors (age, gender, education, DHL). In addition to statistical significance, effect sizes (Cohen’s d) were calculated to evaluate the magnitude of change in Cancer Awareness scores. According to conventional benchmarks, a Cohen’s d of 0.2 is considered a small effect, 0.5 a medium effect, and 0.8 a large effect (27, 28). Analyses were conducted on complete cases only who were linked through their participant IDs from post to pre usage data. We also assessed selection effects by comparing baseline characteristics between responders and non-responders (Age, Age Category, education, DHL category, eHEALS) from the pre usage participants, which are presented in Supplementary File in detail.

### Ethical considerations

The BUMPER pilot study received ethics approval by the University of Bremen (Application No. 2023–10). Participants gave informed consent and were assured of confidentiality and anonymity in published results.

## Results

### Participant characteristics

A total of 328 participants completed the pre-usage survey, with 77 proceeding to the post-usage phase. Among the 251 individuals who did not participate further, 42 provided feedback regarding their reasons for disengagement. The primary barrier was technical difficulties—specifically not receiving the invitation or encountering installation issues—reported by 83% (35/42) of respondents. Time constraints accounted for 5% (2/42), while lack of interest and privacy concerns each represented 2% (1/42). Baseline distributions and summary statistics for responder’s vs. non-responders are provided in Supplementary Tables S1, S2.

### Demographics and health-related characteristics

At pre-usage, participants had a mean age of 44.7 years (SD = 18.2) and a median of 45. The age distribution included 39% young adults, 36% middle-aged adults, and 20% older adults. The sample was predominantly female (74%), with males comprising 26%. Geographically, the largest proportions were from Portugal (27%), Spain (20%), and Slovenia (20%), followed by Germany and Hungary (each 12%). Educational attainment was relatively high, with 34% holding master’s degrees, 21% bachelor’s, and 12% doctoral degrees. Most reported no chronic health conditions (86%), no history of cancer (90%), and low rates of mental health or autoimmune conditions (each 4%).

In the post-usage group, demographic patterns were largely consistent. However, the representation of older adults increased slightly (from 20 to 24%), and there was a modest decline in the proportion of female participants (from 74 to 66%). Hungary had the highest geographic representation post-usage (25%), while Portugal, Slovenia, and Spain showed slight shifts in relative proportions. Among the 77 participants surveyed after usage, 90% were using Android devices, while 10% were using iOS devices. Table 2 below shows the descriptive statistics for demographics and other variables. A small ‘Other European countries’ group is also shown and was retained given the EU wide scope of the app.

**Table 2:** Demographic, device, and health-related characteristics of participants at pre-usage (*n* = 328) and post-usage (*n* = 77).

Category	Subcategory/statistic	Pre usage frequency/statistic (n = 328)	Pre usage (%)	Post usage frequency/statistic (n = 77)	Post usage (%)
Age	Mean ± SD	44.7 ± 18.2	–	44.4 ± 20.5	–
	Median	45	–	44	–
	Young Adults (18–40 years)	128	39	30	36
	Middle-Aged Adults (41–60 years)	119	36	28	34
	Older Adults (≥61 years)	65	20	20	24
	Unknown	16	5	5	6
Gender	Female	242	74	51	66
	Male	86	26	26	34
Country	Portugal	88	27	18	23
	Spain	67	20	13	17
	Slovenia	65	20	14	18
	Germany	40	12	8	10
	Hungary	38	12	19	25
	Cyprus	15	5	3	4
	Other (European countries)^**^	15	4	2	3
Education	Doctoral Degree	39	12	7	9
	Master’s Degree	113	34	25	32
	Bachelor’s Degree	70	21	19	25
	Vocational/Technical Training	24	7	6	8
	Upper Secondary Education	44	13	12	16
	Lower Secondary Education	9	3	1	1
	Primary Education	4	1	–	–
	Other	25	8	7	9
Self-Reported Health Conditions	None	282	86	67	87
	Mental Health	12	4	3	4
	Chronic Illness/Autoimmune	12	4	2	3
	Hearing Impairment	7	2	2	3
	Visual Impairment	6	2	1	1
	Learning Difficulty	6	2	1	1
	Physical Disability	3	1	1	1
Cancer History	No	295	90	66	86
	Yes	32	10	11	14
	Prefer Not to Say	1	0	–	–
Family Cancer History	Yes	263	80	56	73
	No	62	19	20	26
	Prefer Not to Say	3	1	1	1
Mobile Device Test	Android	–	–	69	90
	iOS	–	–	8	10

*Percentages may not sum to 100% due to rounding. ** Other European countries = respondents residing in EU/EEA countries not listed individually.

### Digital health literacy (DHL)

**Figure 1:**
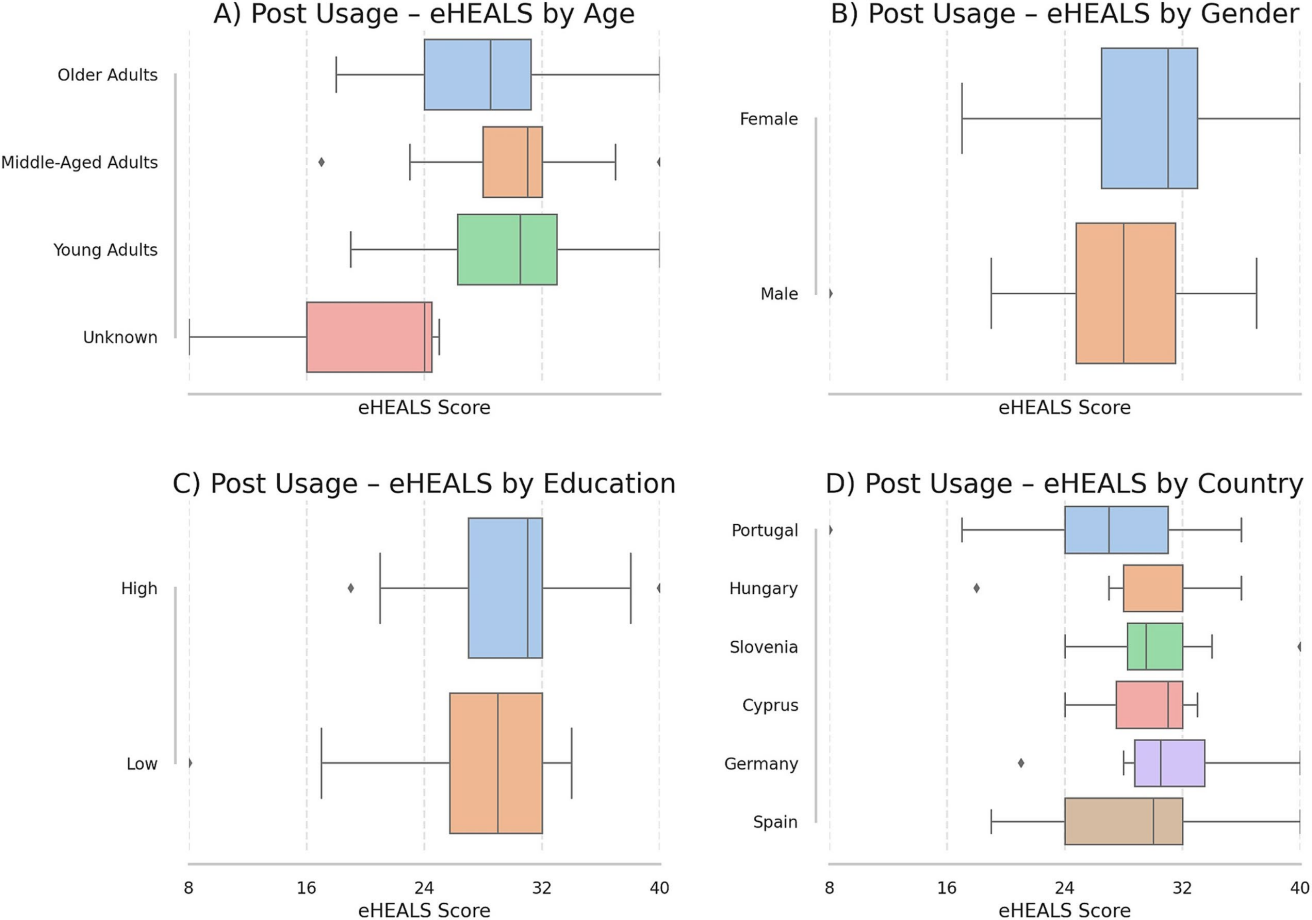
Post-usage DHL scores. This figure presents the distribution of DHL scores across different socio-demographic categories, represented in four boxplots. **(A)** Illustrates the DHL scores for four distinct age categories: young adults, middle-aged adults, older adults, and age not specified (unknown). **(B)** Shows the scores by biological sex, comparing women and men. **(C)** Displays DHL scores based on education level (low: no formal education, primary, lower or upper secondary; high: trade/technical/vocational, bachelor’s degree, master’s degree, and doctorate). **(D)** Compares the DHL scores by country, reflecting the various countries represented in the dataset.

DHL scores are reported only for participants who completed the post-usage survey. The overall mean was 29.3 (SD 5.6). By age group, middle-aged adults had the highest mean 31.5 (SD 4.3), followed by young adults 29.4 (SD 5.9); seniors averaged 28.6 (SD 4.9). Females demonstrated higher DHL 30.1 (SD 5.5) than males 27.5 (SD 5.5). By education, participants with an advanced degree averaged 32.8 (SD 5.7). Figure 1 displays post-usage box plots of DHL scores.

### App usage pattern

Table 3 presents the patterns of app use in terms of both the duration per use and the frequency of usage. Most participants engaged with the app for 5 to 15 min per session (61%), while fewer users reported longer usage times of 16 to 30 min (10%) or 31 to 60 min (4%). Regarding frequency, most respondents used the app either a few times a week (46%) or once every 7 days (43%), with only a small proportion (11%) using it daily.

**Table 3:** App usage characteristics: time per use and frequency among post-usage survey participants (*n* = 77).

App Time per Use			App Usage Frequency		
Category	Count	Percentage (%)	Category	Count	Percentage (%)
Less than 5 min	21	25	Daily	9	11
5 to 15 min	51	61	Few times a week	38	46
16 to 30 min	8	10	Once in 7 days	36	43
31 to 60 min	3	4			

### SUS score descriptives and interpretation

SUS yielded a mean score of 62.56 (SD = 16.9) among post-usage participants (n = 77), with scores ranging from 30 to 100. The median score was 65, and the interquartile range (50.0–72.5) indicated moderate variability, with 50% of participants scoring between 50 and 72.5. While the overall mean fell below the conventional SUS benchmark of 68 (indicating “average” usability), 27.3% of users scored ≥72.5, reflecting a subset of participants who perceived the app as above-average in usability. According to SUS interpretation scales, a mean score of 62.56 falls within the ‘D’ grade range (51.7–62.6), corresponding to the 15th–34th percentile and described as “OK” in terms of usability. This classification indicates marginal acceptability, with users likely to be detractors (users unlikely to recommend the app to others).

### SUS subgroup analysis

**Figure 2:**
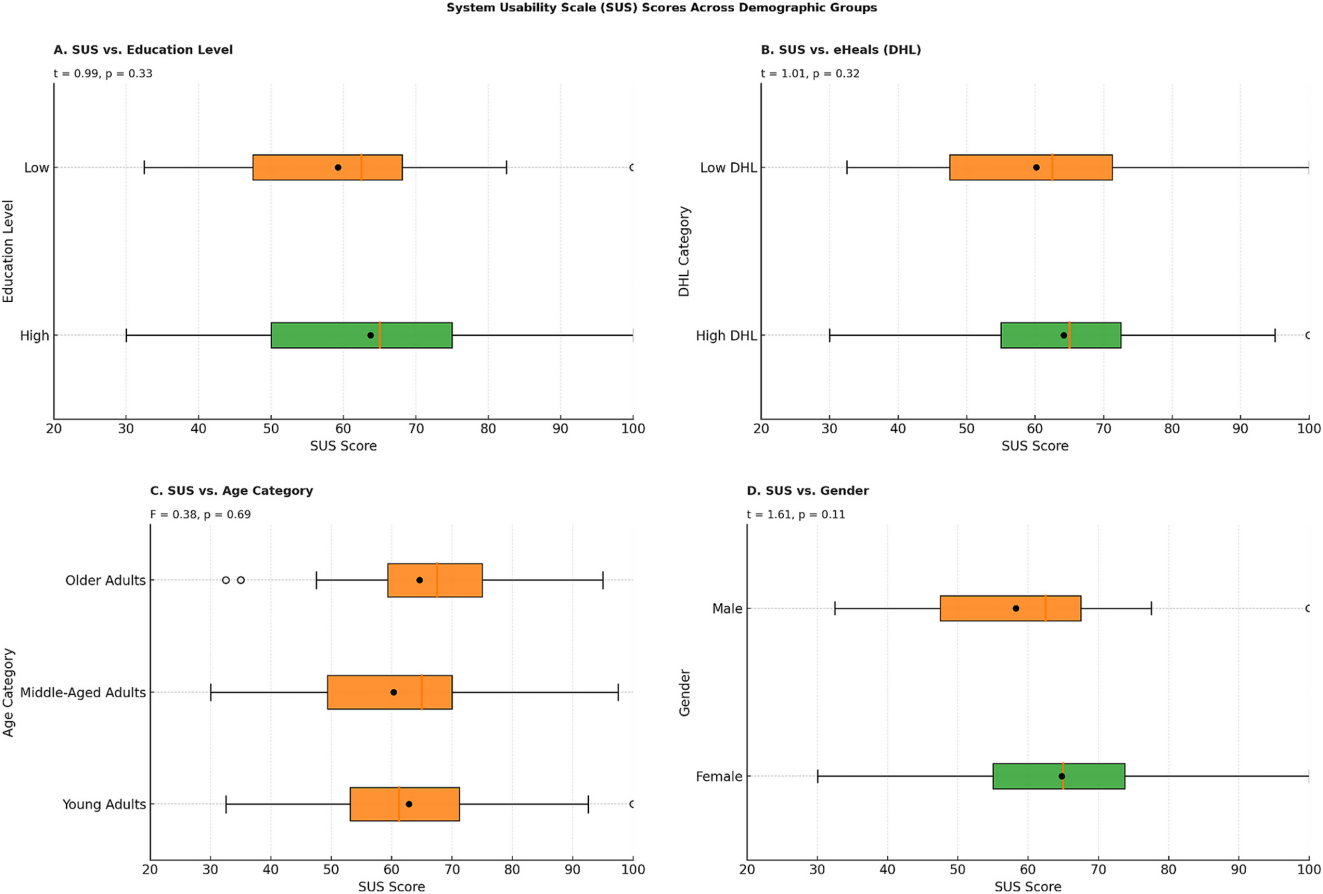
Distribution of system usability scale (SUS) scores by participant demographics among post-usage survey respondents (n = 77). Panels show SUS scores stratified by **(A)** education level, **(B)** digital health literacy, **(C)** age group, and **(D)** gender.

An independent samples t-test was conducted to examine differences in SUS scores across binary categorical variables. The results indicated no significant differences in SUS scores between male and female participants (t = 1.61, *p* = 0.11), high and low education groups (t = 0.99, *p* = 0.33), or low and high DHL groups (t = −1.01, *p* = 0.32). Additionally, a one-way ANOVA was performed to assess the effect of categorical independent variables with more than two groups on SUS scores. The results revealed no significant differences in SUS scores across age groups (*F* = 0.25, *p* = 0.86), mobile device type (*F* = 0.56, *p* = 0.64), app usage frequency (*F* = 0.64, *p* = 0.53), or app time per use (*F* = 0.88, *p* = 0.46). Collectively, these findings suggest that none of the examined demographic or usage-related factors were significantly associated with perceived usability. Descriptive trends (Figure 2) highlighted marginal differences in median SUS scores: older adults had the highest median (median = 67.5, mean = 64.6), followed by middle-aged (median = 65.0, mean = 60.3) and young adults (median = 61.3, mean = 62.8); high education (median = 65.0, mean = 63.7) and high DHL groups (median = 65.0, mean = 64.2) slightly outperformed their counterparts, and females (median = 65.0, mean = 64.8) reported higher scores than males (median = 62.5, mean = 58.3).

### Cancer awareness score

**Figure 3:**
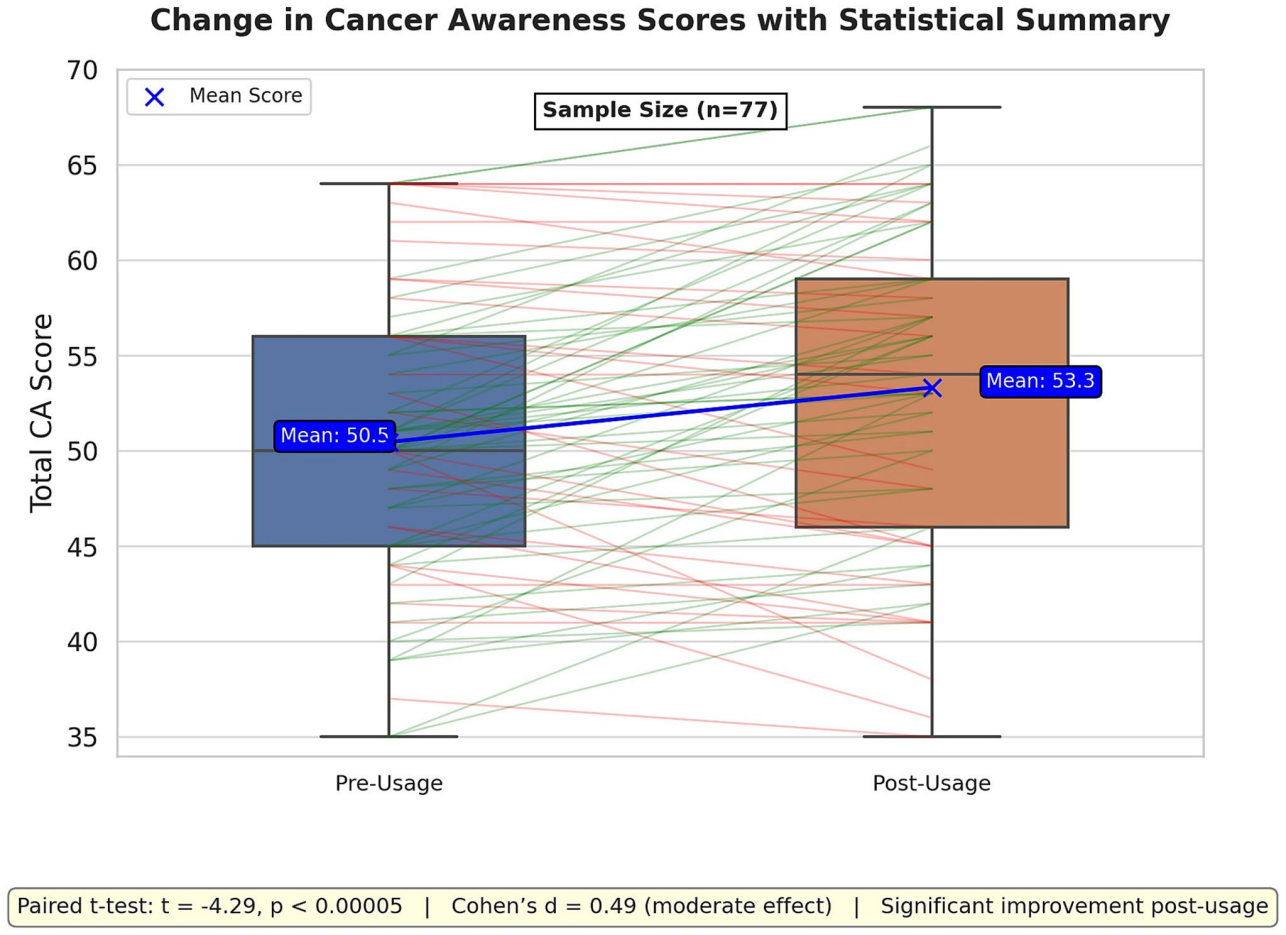
This figure compares cancer awareness (CA) scores before and after app usage using box plots and individual score change lines. Green lines indicate score increases, while red lines show decreases. The mean scores increased from 50.5 (pre-usage) to 53.3 (post-usage), as highlighted by the blue mean trend line.

Descriptive statistics revealed that the mean awareness score before usage was 50.5 (SD = 7.5, N = 77), while the mean score after usage increased to 53.3 (SD = 8.4, N = 77). A paired t-test was conducted to assess the impact of the cancer prevention app on participants’ awareness scores before and after usage. The results indicated a statistically significant increase in awareness scores post-usage (t = −4.29, *p* < 0.001), suggesting that the app had an effect on improving cancer-related knowledge. The effect size (Cohen’s d = 0.49) suggests a moderate impact of the intervention. The boxplot (Figure 3) visualization further supports these findings, showing an upward shift in awareness scores after app usage.

### Cancer awareness score subgroup analysis

**Figure 4:**
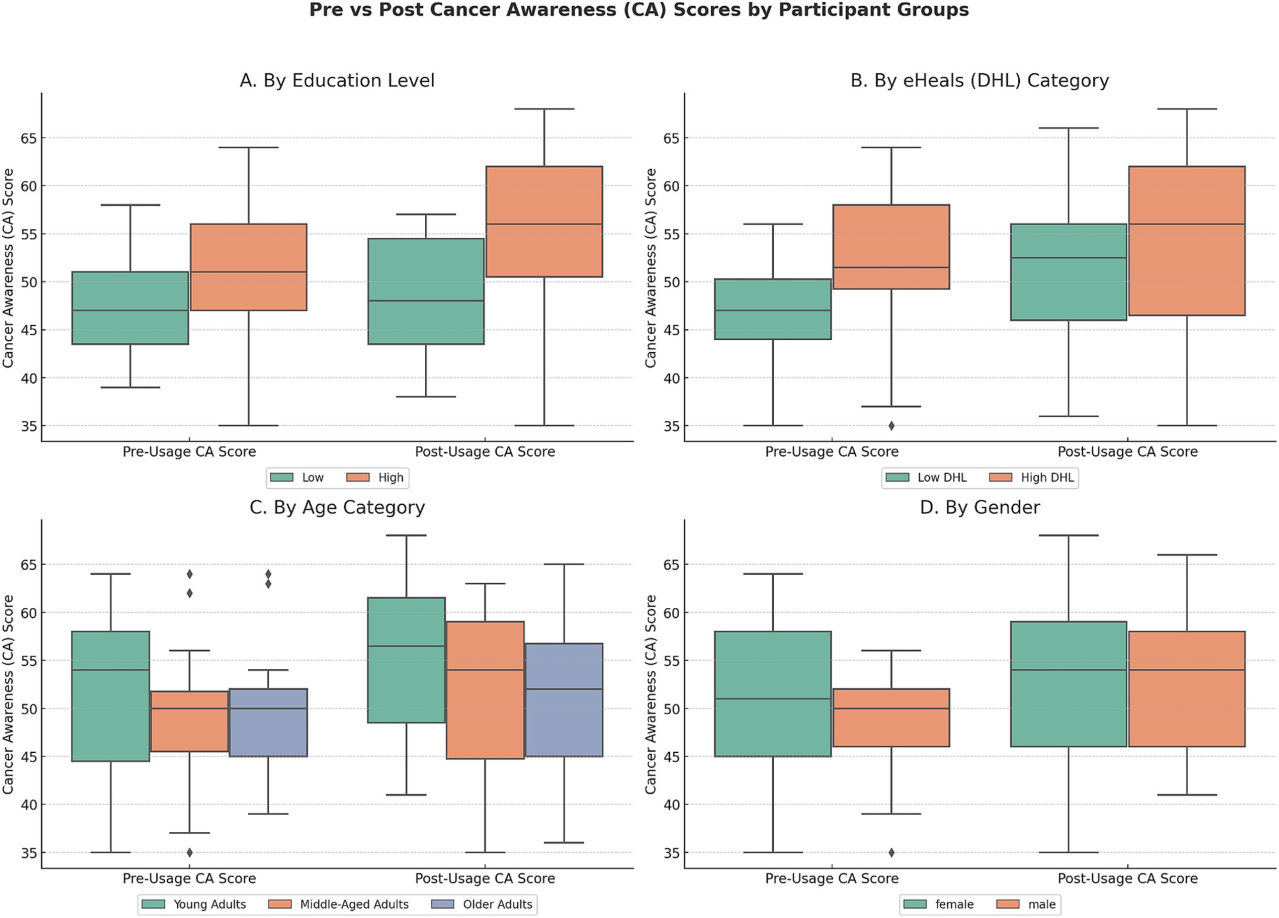
Comparison of pre- and post-usage cancer awareness (CA) scores across participant groups. This figure illustrates changes in CA scores before and after app usage, stratified by Education Level, eHealth Literacy (DHL Category), Age Category, and gender.

Figure 4 presents pre- and post-intervention CA scores across participant subgroups based on education level, DHL, age category, and gender. A general upward trend in CA scores post-usage was observed across most groups. Paired sample t-tests indicated statistically significant improvements in CA scores across several subgroups: participants with high education (t = −4.82, *p* < 0.001), low DHL (t = −4.07, *p* = 0.001), high DHL (t = −2.19, *p* = 0.033), females (t = −2.37, *p* = 0.021), males (t = −2.09, *p* = 0.040), young adults (t = −2.83, *p* = 0.009), and middle-aged adults (t = −3.18, *p* = 0.003), while no significant change was noted for older adults (t = −1.13, *p* = 0.266) or participants with low education (t = −0.68, *p* = 0.503). In terms of effect sizes, with high education showing a medium-to-large effect (d = 0.64), low DHL a large effect (d = 0.73), high DHL a small-to-medium effect (d = 0.32), males a large effect (d = 0.80), females a small-to-medium effect (d = 0.33), young adults a large effect (d = 0.71), and middle-aged adults a medium effect (d = 0.47). Additional analyses by self-reported app usage patterns indicated that changes in CA scores did not differ significantly across frequency groups (daily, few times a week, once per week; *F* = 0.19, *p* = 0.83) or across duration of use (<5 min, 5–15 min, 16–30 min, 31–60 min; *F* = 0.28, *p* = 0.84). This indicates that observed improvements were not systematically greater among participants who reported longer or more frequent interactions with the app.

## Discussion

This study evaluated the EU Mobile App for Cancer Prevention across six European countries (Cyprus, Germany, Hungary, Portugal, Slovenia, Spain) as part of the BUMPER project (8). The app demonstrated moderate usability, aligning with common benchmarks reported in the literature (24, 29). Post-usage cancer awareness scores improved among participants completing both assessments, highlighting its potential efficacy in raising awareness, consistent with previous evaluations of digital health interventions (30, 31). While all demographic groups benefited, older adults (≥55 years) and individuals with lower education showed slightly smaller gains, echoing known disparities in DHL and technology adoption among older populations (32, 33). However, no major disparities were observed by sex or country.

After a seven-day usage period, the app led to a notable increase in cancer awareness, with the mean awareness score rising from 50.5 to 53.3. This outcome demonstrates the app’s potential as an effective educational tool. With a moderate effect size (Cohen’s d = 0.49), the improvement aligns with the objectives outlined by the ECAC and EBCP, emphasizing the importance of health literacy in reducing cancer incidence (7, 34). The modest magnitude of this effect indicates that while the app successfully conveys information on cancer risk factors and prevention, enhancements such as interactive content or extended user engagement could achieve more substantial and lasting impacts (35).

The findings are consistent with prior evidence from mHealth interventions focused on oncology and cancer-related health literacy. For instance, Westerlinck et al. reported that over two-thirds of oncology patients or their family members perceived a cancer risk mobile application as beneficial, with more than half indicating a willingness to modify health behaviors findings suggestive of successful awareness promotion (36). Similarly, Graetz et al. found that an oncology support application, when used during chemotherapy, was associated with a reduction in medical office visits in a randomized controlled trial; however, user engagement was impeded by usability concerns, time limitations, and limited interest among some participants (37). In contrast to these patient oriented tools, the EU Mobile App for Cancer Prevention is designed for use by the general population, yet it encounters comparable challenges in usability. Collectively, these studies highlight the importance of iterative design improvements, the integration of tailored health literacy strategies, and the provision of extended engagement opportunities to optimize the preventive impact of mHealth solutions in cancer prevention and treatment.

Subgroup analysis suggests that cancer awareness improved across diverse demographic and DHL groups, with particularly notable gains among users with higher education, lower DHL, and younger age. Interestingly, individuals with low DHL experienced greater gains than their high DHL counterparts, highlighting the app’s value for users initially having limited digital health competencies (12, 38). However, the absence of significant improvements among older adults and those with lower educational attainment underscores persistent barriers related to technological familiarity or health literacy challenges (39–41). These findings emphasize the importance of tailored mHealth interventions to support these populations effectively (42, 43).

The SUS score of 62.56 (SD = 16.87) places the app in the “OK” usability range (D-grade, 15th–34th percentile), indicating significant usability challenges, including navigation complexity, inconsistent notifications, and performance variability across devices (24, 44). Such issues were noted across demographic and usage groups, highlighting a universal need for design improvements. It is likely that such improvements are particularly beneficial and important, especially for users with lower DHL.

This study provides valuable insights into digital health interventions for cancer prevention, emphasizing key strengths such as integrating validated quantitative tools (SUS, eHEALS), facilitating comprehensive usability assessment (23, 45). The multinational cohort ensured diversity in DHL, age, and sociodemographic variables, aligning with EBCP equity goals (7). The post-intervention improvements in cancer awareness highlight the app’s public health impact potential.

Several limitations should be considered when interpreting these findings. The smaller post-usage sample (n = 77) compared to the initial cohort (N = 328) limits generalizability. High attrition (76.2%) remains a key limitation; while measured baseline differences between responders and non-responders were not statistically significant and effect sizes were negligible–small, unmeasured factors (e.g., onboarding/technical frictions) may still bias post-usage estimates—planned mitigations include improved invite delivery, simplified onboarding, and DHL-tailored guidance (see Supplementary File). The sample’s demographic skew (66% female; 32% with master’s degrees) might not reflect the broader population accurately, particularly marginalized groups with lower literacy or limited digital access (10). The reliance on self-reported measures, such as eHEALS and cancer awareness scores, introduces potential biases. Moreover, while significant short-term improvements in cancer awareness were observed, stratified analyses showed that these gains did not vary by self-reported usage duration or frequency. This absence of a dose response effect suggests that the observed improvements might reflect a survey effect (i.e., participants recalling or searching for correct answers after the baseline survey) rather than being solely attributable to app engagement. The seven-day intervention period was intentionally chosen for pilot testing feasibility but is insufficient to evaluate changes in health behaviors or sustained awareness. Given this brief exposure and the lack of a control group, causal conclusions about the effect of app usage on cancer awareness cannot be drawn. The brief, seven-day intervention period was insufficient to capture sustained behavioral change, thereby highlighting the need for longitudinal studies to assess the long term effectiveness of the intervention (13, 17, 46). Future research should focus on longitudinal studies assessing the app’s long-term impact on cancer awareness and prevention behaviors, iterative usability testing to address identified design flaws, and targeted recruitment of underrepresented populations, such as rural residents, older adults, males, and those with lower educational attainment, to enhance accessibility and effectiveness.

## Conclusion

The EU Mobile App for Cancer Prevention demonstrates potential in boosting cancer prevention awareness and health literacy across six countries, by empowering users with knowledge about risk factors and prevention. The evaluation revealed moderate usability, indicating a need for design improvements to enhance user experience. Addressing usability and accessibility gaps could transform the app into a scalable cancer prevention tool. The findings highlight that effective digital health interventions must balance usability, equity considerations, and opportunity for impact. Lessons from this initiative can guide future mHealth developments, reinforcing the role of policy-aligned, evidence-based digital tools in reducing cancer burden through informed public health strategies. By aligning with the objectives of the ECAC and EBCP, these improvements may contribute to reducing cancer incidence through inclusive, evidence-based public health education, thereby supporting Europe’s strategy against a rising cancer burden in the population.

## Data Availability

The raw data supporting the conclusions of this article will be made available by the authors, without undue reservation.
