# Supplementary material for: Piloting a cancer awareness app across six European countries: a pre-post study
Source: Front Public Health. 2025 Sep 29;13:1648212. doi: 10.3389/fpubh.2025.1648212 (PMC12515922; doi:10.3389/fpubh.2025.1648212)
Supplement: Supplementary file 1 [file Supplementary_file_1.docx]

Table 1. Reasons for Non Participation in the App Pilot Study

| **Reason** | **Yes Count** | **No Count** | **Yes %** | **No %** |
| --- | --- | --- | --- | --- |
| **I did not receive the invitation or had issues installing the app** | 35 | 7 | 83.3 | 16.7 |
| **I did not find time to use the App** | 2 | 40 | 4.8 | 95.2 |
| **I was not interested in the pilot study** | 1 | 41 | 2.4 | 97.6 |
| **I was concerned about privacy or confidentiality** | 1 | 41 | 2.4 | 97.6 |
| **The survey seemed too long or complicated** | 0 | 42 | 0.0 | 100.0 |

**Scoring Scheme for Cancer Prevention Knowledge Quiz**

This scoring scheme provides an objective assessment, ensuring equal weighting across all quiz sections.

**1. Likert Scale Questions (11 Questions)**

Each question evaluates participants' beliefs regarding cancer risk factors.

**Scoring Criteria:**

- Strongly Disagree: **1 point**
- Disagree: **2 points**
- Not Sure: **3 points**
- Agree: **4 points**
- Strongly Agree: **5 points**

**Score Range:**

- **Maximum:** 55 points (11 questions × 5 points)
- **Minimum:** 11 points (11 questions × 1 point)

**2. Age Related Cancer Risk Question (1 Question)**

Participants select the age group most likely to develop cancer.

**Scoring Criteria:**

- 80’s (Correct Answer): **7 points**
- 70’s: **6 points**
- 60’s: **5 points**
- 50’s: **4 points**
- 40’s: **3 points**
- 30’s: **2 points**
- 20’s: **1 point**
- Cancer unrelated to age (Incorrect Answer): **0 points**

**Score Range:**

- **Maximum:** 7 points
- **Minimum:** 0 points

**3. Most Common Cancers in Men and Women (2 Questions)**

Participants must identify the most common cancers for both genders (rank the three options).

**Scoring Criteria:**

- Correct Answer: **1 point per option**
- Incorrect Answer: **0 points**
- No partial credits are awarded.

**Score Range:**

- **Maximum:** 6 points (6 options × 1 point)
- **Minimum:** 0 points

**Final Score Calculation**

Calculate the final score by summing the scores from all sections:

| **Section** | **Minimum Score** | **Maximum Score** |
| --- | --- | --- |
| Likert Scale Questions (11 questions) | 11 | 55 |
| Age Related Cancer Risk Question (1 question) | 0 | 7 |
| Most Common Cancers in Men and Women Questions (2 questions) | 0 | 6 |
| **Total Possible Score** | **11 points** | **68 points** |

**Summary**

- **Maximum Possible Score:** 68 points
- **Minimum Possible Score:** 11 points

This scoring scheme provides an objective and comprehensive measure of participants' knowledge related to cancer prevention.

**Supplementary Note: Attrition and Baseline Comparability**

**Cohort and attrition:** Of N=324 pre usage respondents, n=77 linked to the post usage survey (responders) and n=247 did not (non responders), yielding attrition = 76.2%.

**Statistical approach:** To assess selection effects, we compared baseline characteristics available in both groups (Age, Age Category, education, DHL category, eHEALS). Categorical variables were tested with Pearson χ² (2 sided) and summarized with bias corrected Cramér’s V as effect size (≈0.10 small, 0.30 moderate, 0.50 large). Continuous variables used Welch’s *t* (unequal variances) with Cohen’s *d* (≈0.20 small, 0.50 medium, 0.80 large); median (IQR) also reported.

**Findings:** No statistically significant baseline differences were observed for Age Category (*p*=0.486; V≈0.00), education (*p*=0.991; V≈0.00), or DHL (*p*=0.113; V≈0.07). Treating Age as a multi level categorical variable also showed no difference (*p*=0.290; V≈0.13). For continuous measures, eHEALS was slightly higher among responders (29.25±5.64 vs 27.98±6.37; *p*=0.096; *d*≈0.21, small), while Age was similar (47.24±16.97 vs 45.91±15.34; *p*=0.548; *d*≈0.08, negligible).

**Table S1. Baseline categorical comparisons (responders vs non responders)**

*Responders = participants who completed the post usage survey and could be linked to their pre usage survey; Non responders = pre usage participants who did not complete the post usage survey. Percentages are within group (column %). Test = Pearson χ² (2 sided); effect size = bias corrected Cramér’s V.*

| **Variable** | **Category** | **Responders n (%)** | **Non responders n (%)** | **χ² p** | **Cramér’s V** |
| --- | --- | --- | --- | --- | --- |
| **Age Category** | Young Adults | 30 (38.96%) | 96 (38.87%) | **0.486** | **0.000** |
|  | Middle Aged Adults | 24 (31.17%) | 91 (36.84%) |  |  |
|  | Older Adults | 20 (25.97%) | 46 (18.62%) |  |  |
|  | Unknown | 3 (3.90%) | 14 (5.67%) |  |  |
| **Education** | High | 57 (74.03%) | 183 (74.09%) | **0.991** | **0.000** |
|  | Low | 20 (25.97%) | 64 (25.91%) |  |  |
| **DHL** | High DHL | 46 (59.74%) | 122 (49.39%) | **0.113** | **0.068** |
|  | Low DHL | 31 (40.26%) | 125 (50.61%) |  |  |

*Abbreviation*: DHL, Digital Health Literacy (categorical).

**Table S2. Baseline continuous comparisons (responders vs non responders)**

*Summaries are mean (SD) and median (IQR). Test = Welch’s t (unequal variances, 2 sided); effect size = Cohen’s d (≈0.20 small, 0.50 medium, 0.80 large).*

| **Variable** | **Responders mean (SD)** | **Responders median (IQR)** | **Non responders mean (SD)** | **Non responders median (IQR)** | **Welch *t* p** | **Cohen’s *d*** |
| --- | --- | --- | --- | --- | --- | --- |
| **eHEALS** | 29.25 (5.64) | 30.00 (6.00) | 27.98 (6.37) | 28.00 (8.00) | **0.096** | **0.205** |
| **Age** | 47.24 (16.97) | 44.50 (29.50) | 45.91 (15.34) | 46.00 (25.00) | **0.548** | **0.085** |

Sample sizes: Pre usage N=324; Responders n=77; Non responders n=247; Attrition = 76.2%.

**Missing data and attrition analyses:** Pre–post analyses were conducted on complete cases (responders with both time points); no imputation was performed for missing post usage data. To evaluate attrition related selection, baseline characteristics were compared between responders and non responders using Pearson’s χ² with bias corrected Cramér’s V for categorical variables and Welch’s *t* with Cohen’s *d* for continuous variables, reporting column percentages and median (IQR) in addition to mean (SD).

**High attrition and potential bias:** The drop from N=324 to n=77 (76.2%) is substantial. Baseline comparisons did not identify significant differences between responders and non responders for Age Category, education, or DHL, and eHEALS showed a small, non significant trend toward higher literacy among responders (*p*=0.096; *d*≈0.21). These results suggest limited measurable baseline imbalance on available variables; however, unmeasured factors particularly onboarding/technical frictions may still have selectively excluded some participants, potentially biasing post usage estimates toward those who could install and use the app successfully. Planned mitigations include more reliable invite delivery, simplified onboarding, and DHL tailored guidance to minimize selective dropout.
